# Repetitive Immunization Enhances the Susceptibility of Mice to Peripherally Administered Prions

**DOI:** 10.1371/journal.pone.0007160

**Published:** 2009-09-25

**Authors:** Juliane Bremer, Mathias Heikenwalder, Johannes Haybaeck, Cinzia Tiberi, Nike Julia Krautler, Michael O. Kurrer, Adriano Aguzzi

**Affiliations:** 1 Institute of Neuropathology, Department of Pathology, University Hospital of Zurich, Zurich, Switzerland; 2 Institute of Pathology, Kantonsspital Aarau, Aarau, Switzerland; Université de Toulouse, France

## Abstract

The susceptibility of humans and animals to prion infections is determined by the virulence of the infectious agent, by genetic modifiers, and by hitherto unknown host and environmental risk factors. While little is known about the latter two, the activation state of the immune system was surmised to influence prion susceptibility. Here we administered prions to mice that were repeatedly immunized by two initial injections of CpG oligodeoxynucleotides followed by repeated injections of bovine serum albumin/alum. Immunization greatly reduced the required dosage of peripherally administered prion inoculum necessary to induce scrapie in 50% of mice. No difference in susceptibility was observed following intracerebral prion challenge. Due to its profound impact onto scrapie susceptibility, the host immune status may determine disease penetrance after low-dose prion exposure, including those that may give rise to iatrogenic and variant Creutzfeldt-Jakob disease.

## Introduction

Prion diseases, or transmissible spongiform encephalopathies (TSE), are infectious neurodegenerative conditions that typically lead to cognitive and motor dysfunction [1], [2]. With the exception of rare chronically presymptomatic carriers [3], prion diseases are progressive, fatal, and presently incurable. Prions propagate during the course of the disease and form aggregates containing PrP^Sc^, a misfolded, beta-sheet-rich isoform of the cellular prion protein PrP^C^, which is encoded by the *PRNP* gene [4], [5]. Typical neuropathological features include neuronal loss, astrogliosis and spongiform changes [1], [2].

In many instances, prion infections are acquired iatrogenically or by oral uptake of prion-contaminated food [6]. For example, bovine spongiform encephalopathy (BSE) has been transmitted within cattle populations by prion-tainted meat and bone meal [7]. The ritual consumption of deceased relatives is the attributed cause of the epidemic cases of Kuru in Papua New Guinea with incubation periods exceeding 50 years [8]. Variant Creutzfeldt-Jakob disease (vCJD) is believed to be caused by the consumption of beef contaminated with BSE prions, as strongly suggested by epidemiological, biochemical and neuropathological analyses as well as transmission studies [9], [10], [11], [12], [13], [14], [15]. The association between BSE and vCJD, together with the high number (at least 190,000) of BSE infected cows, mainly in the UK in the 1980s and early 1990s, suggest a highly prevalent exposure of the European population to BSE prions and have raised fears of an upcoming vCJD epidemic. Fortunately, the incidence of vCJD remained disproportionally low: roughly 200 human individuals succumbed to vCJD until now and the incidence is declining.

The pivotal factors determining susceptibility to prion disease of the exposed population remain largely unknown. Presence of the cellular prion protein is certainly essential, since the absence of PrP prevents disease in mice inoculated peripherally or intracerebrally with prions [16], [17], yet PrP^C^ expression alone is not sufficient to sustain prion replication [18], [19]. Intensive research has been carried out to identify further risk factors, the major one being the Met/Val polymorphism at codon 129 of the *PRNP* gene [20]. Almost all vCJD patients to date have been found to be homozygous for Met at this codon [5], [21], [22] and heterozygosity at codon 219 (219^Glu/Lys^) is associated with decreased risk to develop sCJD [23].

Much less is known about any non-genetic risk factors. Analyses of epidemiological data of different prion diseases, including scrapie, BSE, and vCJD suggested that the risk for TSEs may be age-dependent [24], [25], [26], but no further non-genetic risk factors are known. It has been known for a long time that injections of the immunomodulatory glucocorticosteroid prednisone prolonged incubation time after intraperitoneal, but not intracerebral, injection of scrapie-infected brain homogenate [27], suggesting that the lymphoid system acts as a “Trojan horse” instead of a defense mechanism during scrapie pathogenesis. Indeed, prion replication occurs in lymphoid tissues long before neuroinvasion and subsequent detection in the central nervous system (CNS) [6].

Within secondary lymphoid organs, follicular dendritic cells (FDCs) play a key role in peripheral prion replication and disease pathogenesis. FDCs located within germinal centers express high levels of PrP^C^ and accumulate PrP^Sc^ [28]. Maturation and maintenance of FDCs depend on tumor necrosis factor alpha (TNF-α) and lymphotoxins (LT-α and LT-β). Mice lacking TNF-α, complement components and their receptors, LT-α, LT-β, or LT-β receptor are partially resistant to peripheral prion infection [29], [30]. Mice treated with an inhibitor of LT-β-receptor signaling (LT-βR-Ig) displayed a reversible dedifferentiation of FDCs. This leads to a decreased susceptibility to orally or intraperitoneally administered prions [31], [32], [33]. In inflammatory conditions, accordingly, extravasating immune cells enable prion replication at the sites of chronic inflammation [34], [35] and may even lead to prion excretion [36]. Newborn mice whose immune system has not fully matured were shown to display a strongly reduced susceptibility to extracerebrally administered prions [37]. The increase in susceptibility with age correlated with the immunocytochemical detection of PrP^C^ on maturing FDCs [38].

Mature FDCs are unlikely to transport prions to peripheral nerve terminals. However, the relative distance between FDCs and peripheral nerves [39], and PrP^C^ expression in the peripheral nervous system [40] determine prion neuroinvasion efficiency and onset of terminal disease. Also, sympathectomy delays or prevents scrapie following intraperitoneal prion inoculation [41]. The precise mechanisms of prion transport from prion replicating germinal centers to peripheral nerves remain elusive. While germinal center B-cells do not appreciably contribute to intrasplenic prion trafficking [42], [43], the identity of the crucial actuators of trafficking, be they hematopoietic or stromal cells, or even subcellular particles, remains to be determined. The presence of antigen presenting cells (APCs) was described to be a prerequisite for lympho- and neuroinvasion after peripheral prion infection [44], [45], [46], [47], [48], although others have questioned their importance [49].

It was reported that repeated administration of CpG-containing oligodeoxynucleotides (CpG-ODN) decreases the susceptibility to prions [50]. Since CpG-ODN activate the Toll-like receptor 9 (TLR9), these surprising findings were interpreted as evidence that activation of the innate immune system may be protective against prions. However, we subsequently found that repeated injections of CpG-ODN dramatically compromise morphology and functionality of murine lymphoid organs [51]. Due to the mechanisms discussed above, the immunosuppressive properties of CpG-ODN are much more likely to account for the reported antiprion effects than any conjectured immune activation [52].

Since the immune system has an essential role in peripheral prion pathogenesis, we hypothesized that stimulation of the immune system might increase the susceptibility towards peripherally administered prions [52]. To test this, laboratory mice (C57BL/6) were confronted with a short-term challenge of CpG-ODN followed by repeated immunization with bovine serum albumin (BSA) and alum. This experimental protocol was devised with the goal to broadly stimulate both the innate and the adaptive components of the immune system for a protracted period of time. Histological, hematological analysis as well as serum markers identified changes indicative of moderate stimulation of the immune system. By serial dilutions of the inocula, we could indeed identify a dose of peripherally administered prions causing scrapie in 50% of immunized mice whereas control mice remained healthy. In contrast, repetitive immunization did not affect prion susceptibility to intracerebrally administered prions.

## Results

### Stimulation of the immune system by repetitive immunization

**Figure 1:**
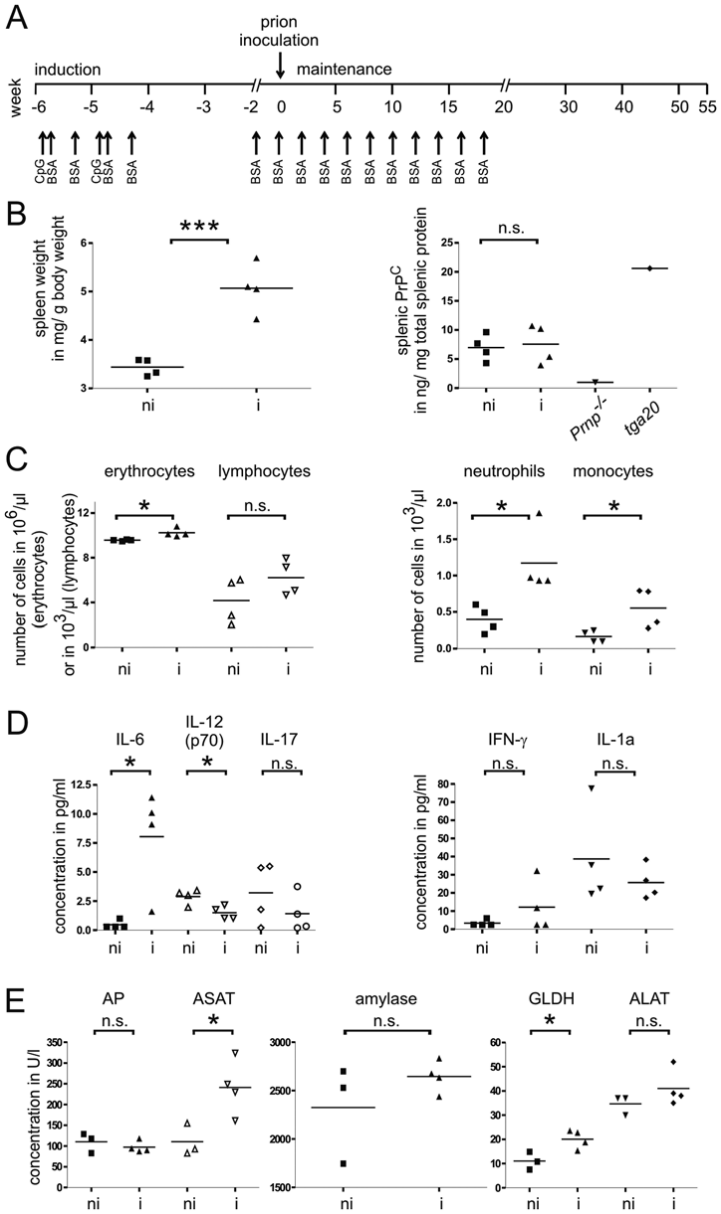
Repetitive immunization of wild-type mice. (A) Treatment scheme for immunization. Arrows denote the time points of injections of CpG oligodeoxynucleotides and bovine serum albumin (BSA). Treatment included an induction (−6 to −4 weeks) and a maintenance phase (−2 to +18 weeks). (B–E) Selected immunized (i) and non- immunized mice (ni) were sacrificed at the time point of prion inoculation. (B) Relative spleen weight and splenic PrP^C^ levels as determined by ELISA. Spleen derived from a PrP^C^ deficient mouse (*Prnp^−/−^*) served as negative, spleen from a PrP^C^ overexpressing mouse (*tg*a*20*) as positive control. Hematological cell counts (C), serum levels of selected cytokines (D), and blood chemistry (E) (AP: alkaline phosphatase; ASAT: aspartate amino transferase; GLDH: glutamate dehydrogenase; ALAT: alanine amino transferase) showed mild polyglobulia and leukocytosis, as well as mild liver damage in repetitively immunized mice. Unpaired t-tests: (*) p<0.05; (***) p = 0.001; (n.s.) not significantly different.

Starting 6 weeks prior to prion inoculation, wild-type mice (C57BL/6) were immunized and repeatedly boosted with generic antigens unrelated to prions, with the goal of achieving sustained activation of immune cells and activation-related morphological changes within lymphoreticular organs. A mixture of CpG-ODN, bovine serum albumin (BSA) and alum was repeatedly injected intraperitoneally during a period of two weeks (Fig. 1A). For the following two weeks, treatment was suspended in order to prevent damage to secondary lymphoid organs as observed following long-term administration of CpG-ODN (data not shown). Mice were then injected every other week for 20 weeks with BSA/alum.

At the time point of prion inoculation, selected animals were sacrificed to evaluate organs and blood for histological, cytological, and biochemical evidence of immune stimulation or inflammation, respectively. When compared to age and gender-matched non-immunized wild-type controls, immunized animals displayed typical features of immune stimulation. Spleens of immunized mice were significantly heavier, yet the concentration of splenic PrP^C^ (weight/weight) remained unchanged (Fig. 1B), indicating that the total amount of splenic PrP^C^ was increased. In peripheral blood, chronic immunization resulted in significantly elevated cell counts of erythrocytes, neutrophils, and monocytes. Lymphocyte counts were marginally increased but did not reach statistical significance (Fig. 1C).

We also investigated levels of various cytokines in sera. IL-6 was elevated in mice with inflammation, whereas IL-12 (p70) was reduced and IL-1α was unaltered. IFN-γ was also increased though not significantly (Fig. 1D), and TNF-α was below detection limit in all animals analyzed (data not shown).

**Figure 2:**
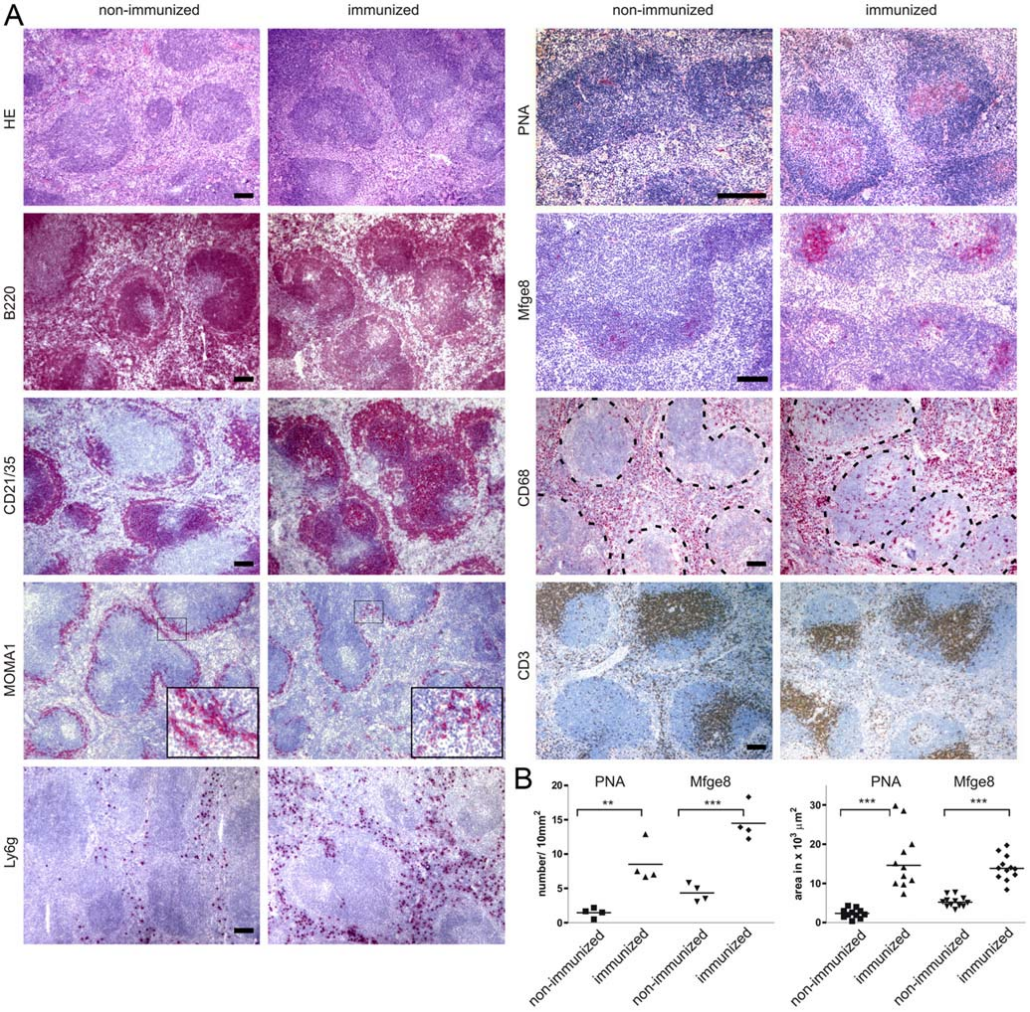
Splenic histology of immunized mice. (A) Spleens of non-immunized and immunized mice were analyzed at the time point of inoculation by histology with hematoxylin-eosin (HE) and immunohistochemistry for B-cells (B220), complement receptors (CD21/35), metallophilic marginal zone macrophages (MOMA-1), granulocytes (Ly6g), germinal center B-cells (PNA), FDCs (Mfge8), macrophages (CD68), and T-cells (CD3). The overall splenic microarchitecture was preserved. Immunized mice showed broader marginal zones, some loosening of splenic MOMA-1^+^ metallophilic marginal zone macrophage festoons and a reduction of the density of MOMA-1^+^ cells compared to non-immunized animals. Following immunization, granulocytes (Ly6g) in the red pulp and macrophages (CD68) in the white pulp (indicated by dashed lines) were increased. (B) Densities and size of PNA^+^ germinal center B-cells and FDC networks (Mfge8) were also increased in immunized mice. Scale bars: 100 µm. Statistics was performed using unpaired t-test, two-tailed.

**Figure 3:**
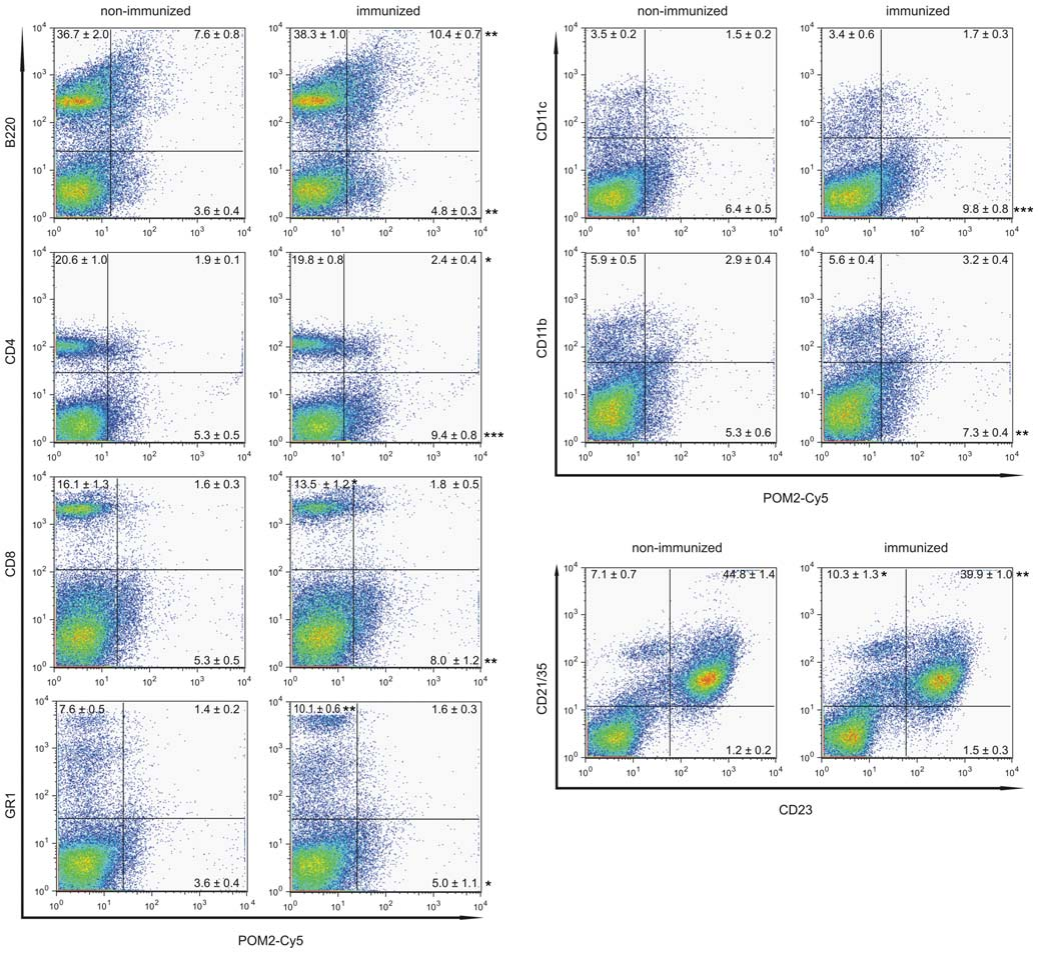
Splenic cell populations in immunized mice. Splenic cells were isolated and co-stained for PrP^C^ and B220, CD4, CD8, GR1, CD11c, or CD11b; alternatively co-staining for CD21/35 and CD23 was performed. Results from representative samples are shown. Four mice per group were analyzed. Numbers in the diagram indicate average percentages of cells ± standard deviation. Statistical analysis was performed using unpaired t-test, two-tailed. Significantly different percentages are labeled in the right lower scattergram with (*) p<0.05; (**) p<0.01; or (***) p<0.001.

Splenic white pulp follicles appeared more densely packed upon immunization (3.78±0.31 and 7.89±2.71 follicles/mm^2^ in histological sections of non-immunized mice and immunized mice, respectively; *p* = 0.029). Immunized mice showed a significant increase in density and size of PNA^+^ germinal centers and Mfge8^+^ follicular dendritic cell networks. In addition, we observed a loosening of MOMA-1^+^ metallophilic macrophage festoons in the marginal zone following immunization as described previously [51] (Fig. 2). Marginal zones appeared broader also in B220 and CD21/35 immunostains. Accordingly, flow cytometry of splenocytes showed a higher percentage of CD21/35^+^CD23^−^ marginal zone B-cells in immunized mice than in controls (Fig. 2 and Fig. 3).

*Mfge8* transcription is not only confined to germinal centers, but also occurs in cells located in the marginal zone and were suggested to represent FDC precursors [53]. However, *in situ* hybridization did not identify any differences in *Mfge8^+^* cells residing within the marginal zones of immunized and control mice (Supplemental Figure S1). Instead, immunization increased the prevalence of splenic GR1/Ly6g^+^ granulocytes, as verified by immunohistochemistry and flow cytometry (Fig. 2 and Fig. 3), and increased the number of CD68^+^ macrophages in splenic white pulp follicles (Fig. 2). In contrast, the frequency of splenic CD11b^+^ and CD11c^+^ cells was unaffected (Fig. 3). Although total splenic PrP^C^ protein concentration was unaltered, flow cytometry revealed a slight yet significant increase in PrP^C^ surface expression by splenic B220^+^ and CD4^+^ cells (Fig. 3).

Histological analysis of mesenteric lymph nodes (MLNs) showed a trend towards increased numbers of lymphoid follicles (non-immunized mice: 6±2.31; immunized mice: 11.75±1.44 follicles/lymph node section, *p* = 0.11; Supplemental Figure S2A). Furthermore, histology showed that immunized mice had a normal architecture of lung, heart, kidney, and pancreas, yet developed lobular hepatitis with loss of hepatocytes and multifocal infiltrates of lymphocytes, macrophages, eosinophils, and neutrophils (Supplemental Figure S2B and data not shown). Hepatocellular damage was confirmed by elevation of the liver enzymes glutamate dehydrogenase (GLDH) and aspartate amino transferase (ASAT; Fig. 1E). Serum amylase and alkaline phosphatase (AP) levels were normal (Fig. 1E), suggesting that there was no damage to the exocrine pancreas and the bile ducts. Finally, immunization induced a mild peritonitis with macrophages and lymphocytes in the peritoneum (Supplemental Figure S2C).

### Increased prion susceptibility in immunized mice

**Figure 4:**
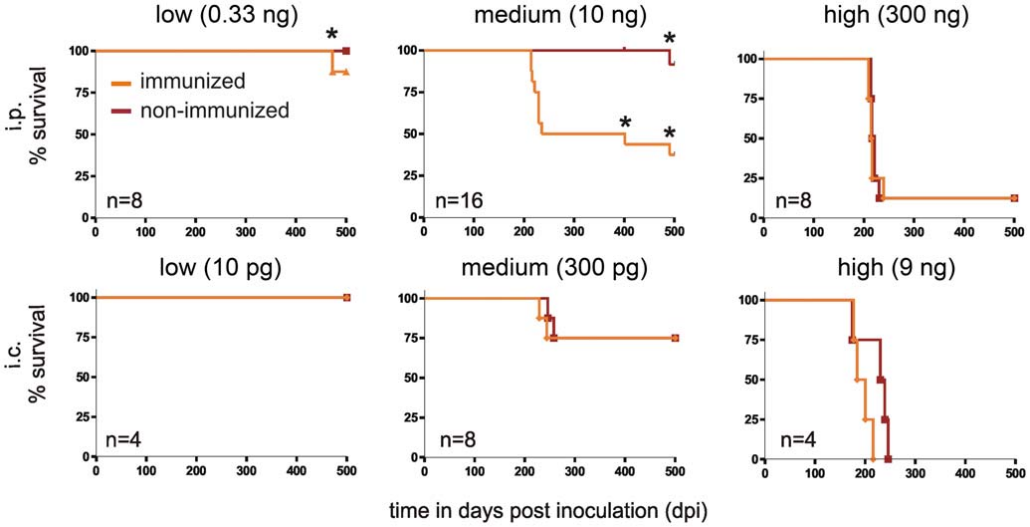
Survival of immunized (i) and non-immunized (ni) mice inoculated intraperitoneally (i.p.) or intracerebrally (i.c.) with prions. While all mice inoculated i.p. or i.c. with a low dose of prions (0.33 ng i.p. or 10 pg i.c. RML6 brain homogenate; left panel) survived until 500 dpi, all mice inoculated i.c. with a high dose of prions (9 ng; right lower panel) died of scrapie. Seven of 8 mice (87.5%) inoculated i.p. with a high dose of prions (300 ng) succumbed to scrapie independently of immunization (right upper panel). In the medium dose group (10 ng i.p. or 300 pg i.c.; medium panels) there was no difference in survival rates in i.c. inoculated mice (75% in both groups). While non-immunized mice inoculated i.p. with a medium dose of prions remained healthy, 50% of the immunized mice developed scrapie. Intercurrent deaths (not scrapie-associated) occurred >400 dpi; time points are marked with a star above the curve. All brains of mice dying without clinical signs of scrapie were tested by Western blot and did not contain PrP^Sc^ (data not shown).

Groups of mice (4≤n≤16) were inoculated intraperitoneally (i.p.) or intracerebrally (i.c.) with low, medium or high doses of prion infectivity as detailed in Fig. 4. Upon i.p. inoculation with 10 ng of RML6 brain homogenate, 8 of 16 immunized mice (50%) succumbed to scrapie at 223±8.2 days post inoculation (dpi). When exposed to the same dose of prions, non-immunized animals (n = 16) remained scrapie-free for >500 dpi (Fig. 4). This difference in survival was highly significant (*p* = 0.0024, Fisher's exact test) and could have two non-exclusive explanations: 1) immunization may increase the accumulation, spread, and/or propagation of prions, or 2) immunization may promote the progression of subclinical infection to clinically overt disease. To study the latter possibility, we investigated non-immunized mice that had received the same dose of peripherally administered prions (10 ng) for subclinical signs of prion infection at 400 dpi. Non-immunized mice did not display PrP^Sc^ in brains and spleens, nor did they show any histological signs of prion disease such as astrogliosis and microglial activation (Figs. 4–6). In addition, we observed no difference in the time intervening between onset of clinical signs and development of terminal scrapie (data not shown). Therefore, repeated immunization of wild-type mice increased the susceptibility to prion diseases by modulating prion accumulation, spread, and/or propagation rather than by simply affecting the onset of clinical signs.

**Figure 5 pone-0007160-g005:**
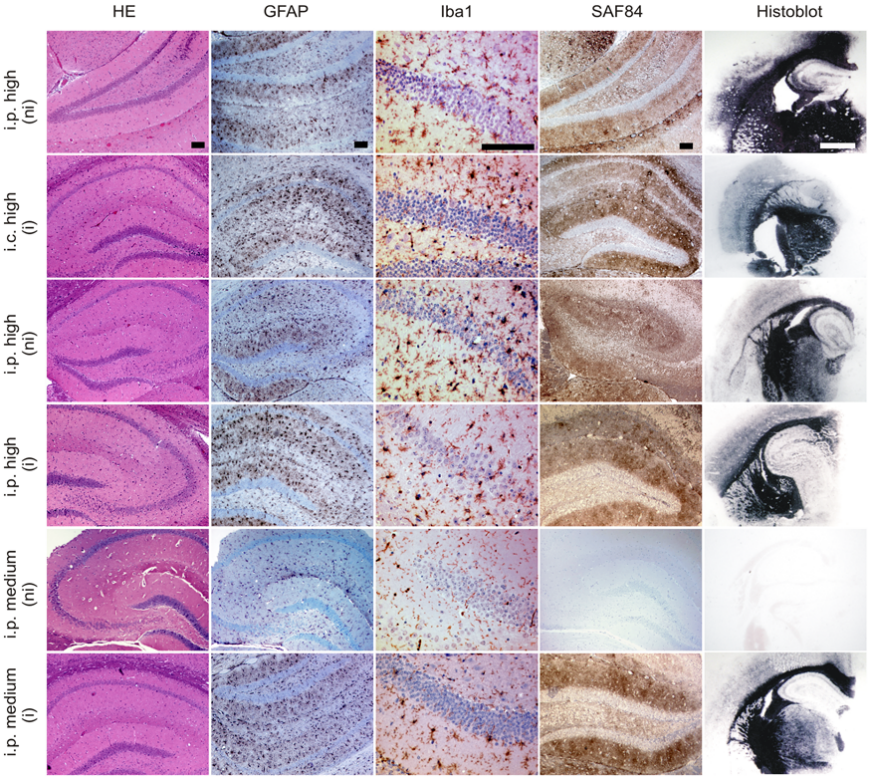
Brain pathology of terminally sick or asymptomatic mice. Immunized (i) and non-immunized mice (ni) inoculated intracerebrally (i.c.) or intraperitoneally (i.p.) with medium or high dose of prions were analyzed by histology. HE stains and immunohistochemistry were performed as indicated: astrocytes (GFAP), microglia (Iba1), PrP deposits (SAF84), showing astrogliosis, microglia activation, and prion protein deposition in terminally sick mice from each of the experimental groups. Histoblot analyses detected PrP^Sc^ in the brains of all terminally sick mice with similar distribution patterns. Non-immunized mice inoculated i.p. with a medium dose of prions remained healthy up to 500 dpi, and displayed much less GFAP^+^ astrocytes and Iba1^+^ microglia cells, and lacked both PrP deposits (SAF84) and PrP^Sc^ (histoblot) at 400 dpi. Scale bars: histoblot = 1 mm; histology = 100 µm.

**Figure 6:**
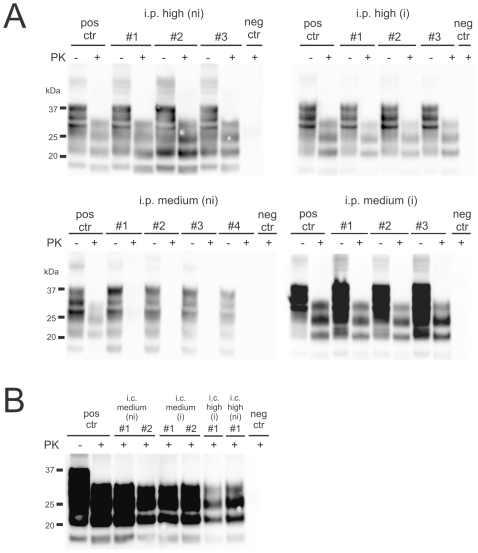
PrP^Sc^ in brains of terminally sick or asymptomatic mice. Immunized (i) and non-immunized mice (ni) inoculated (A) intraperitoneally (i.p.) or (B) intracerebrally (i.c.) with medium or high doses of prions were analyzed by Western blots for the presence of PrP^Sc^. Brain homogenates were analyzed with (+) and without (−) previous proteinase K (PK) treatment as indicated. Homogenate derived from a terminally scrapie-sick mouse served as positive control (pos ctr), and healthy wild-type mouse tissue as negative control (neg ctr), respectively. Molecular weights are indicated on the left side of the blots. All terminally sick mice showed considerable amounts of PrP^Sc^ in the brain. In contrast, non-immunized mice inoculated i.p. with a medium dose of prions [i.p. medium (ni)] lacked PrP^Sc^.

While inducing scrapie in immunized mice, the dose of prion inoculum utilized in the above mentioned experimental series was insufficient to establish infection in non-immunized mice. We therefore wondered what would happen after administration of a dose that would induce a 100% attack rate in non-immunized mice. We inoculated immunized and non-immunized mice i.p. with a 30-fold higher dose of prions (300 ng RML6 brain homogenate). No difference in the percentage of mice succumbing to disease was observed: 7 of 8 mice in both groups (87.5%; Fisher's exact test *p* = 1.0) developed terminal scrapie. This indicates that immunization selectively lowers the size of the minimal infectious dose, while it does not change the course of the disease when larger doses are administered. Accordingly, the incubation times in non-immunized (219±5.7 dpi; n = 8) and in immunized mice (217±10.3 dpi; n = 8) inoculated with the highest dose were similar to those in immunized mice that had received the medium dose (223±8.2 dpi, n = 16; ANOVA *p* = 0.3). Therefore, stimulation of the immune system by repeated immunization does not accelerate the general progression of the disease, but rather renders mice susceptible to amounts of peripherally administered prions that would be innocuous to non-immunized wild-type mice.

We suspected that alterations in lymphoid organs mediate the observed differences in susceptibility. As these events are not thought to be relevant to CNS prion pathogenesis, we expected that the difference in susceptibility would be abolished in immunized versus non-immunized mice inoculated i.c. with prions. The injection of 300 pg RML6 brain homogenate caused disease in 2 of 8 mice (25%) in both groups. Survival curves did not significantly differ (logrank test, *p* = 1). A 30-fold higher prion dose (9 ng RML6 brain homogenate) inoculated i.c. elicited an attack rate of 4 of 4 (100%) in both groups. Although immunization of wild-type mice strongly increased prion susceptibility to i.p. administered prions, prion susceptibility remained unchanged after i.c. inoculation. This emphasizes the decisive role of peripheral immune system components in determining prion susceptibility. Despite the absence of differential susceptibility, there were slight differences in the incubation times at high and medium dose of prion challenge. However, in both instances these differences were not statistically significant (high dose: 9 ng i.c.; non-immunized: 222±32.8 dpi; immunized: 194±17.4 dpi, unpaired t-test *p* = 0.18; medium dose: 300 pg i.c.; non-immunized: 246/258 dpi; immunized: 229/244 dpi).

We then inoculated immunized and naïve mice i.p. with 0.33 ng RML6 homogenate, or i.c. with 10 pg RML6 homogenate. None of these mice (immunized or naive) developed scrapie or neurological signs up to 500 dpi. These data and the absence of astrogliosis and microglia activation in brains of the non-immunized mice (10 ng i.p. at 400 dpi) described above (Fig. 5) argues against any neurotoxic effects of BSA/alum/CpG-ODN.

### PrP^Sc^ distribution patterns and histopathological features in terminally scrapie-sick mice

Brains of all mice developing clinical signs of scrapie were investigated for histological features of prion disease, including astrogliosis, spongiosis, PrP deposition and microglial activation (Fig. 5). There were no differences in these histological hallmarks of scrapie. In contrast, those non-immunized mice (n = 4) inoculated i.p. with prions (10 ng RML6 brain homogenate) that remained healthy lacked any histological features of prion disease at 400 dpi. Although the SAF84 antibody is commonly used to detect PrP aggregates, it is not specific for PrP^Sc^. Therefore, in addition we investigated brains by histoblot (Fig. 5) and immunoblot (Fig. 6). Both techniques demonstrated the presence of PrP^Sc^ in all clinically scrapie-sick mice.

**Figure 7:**
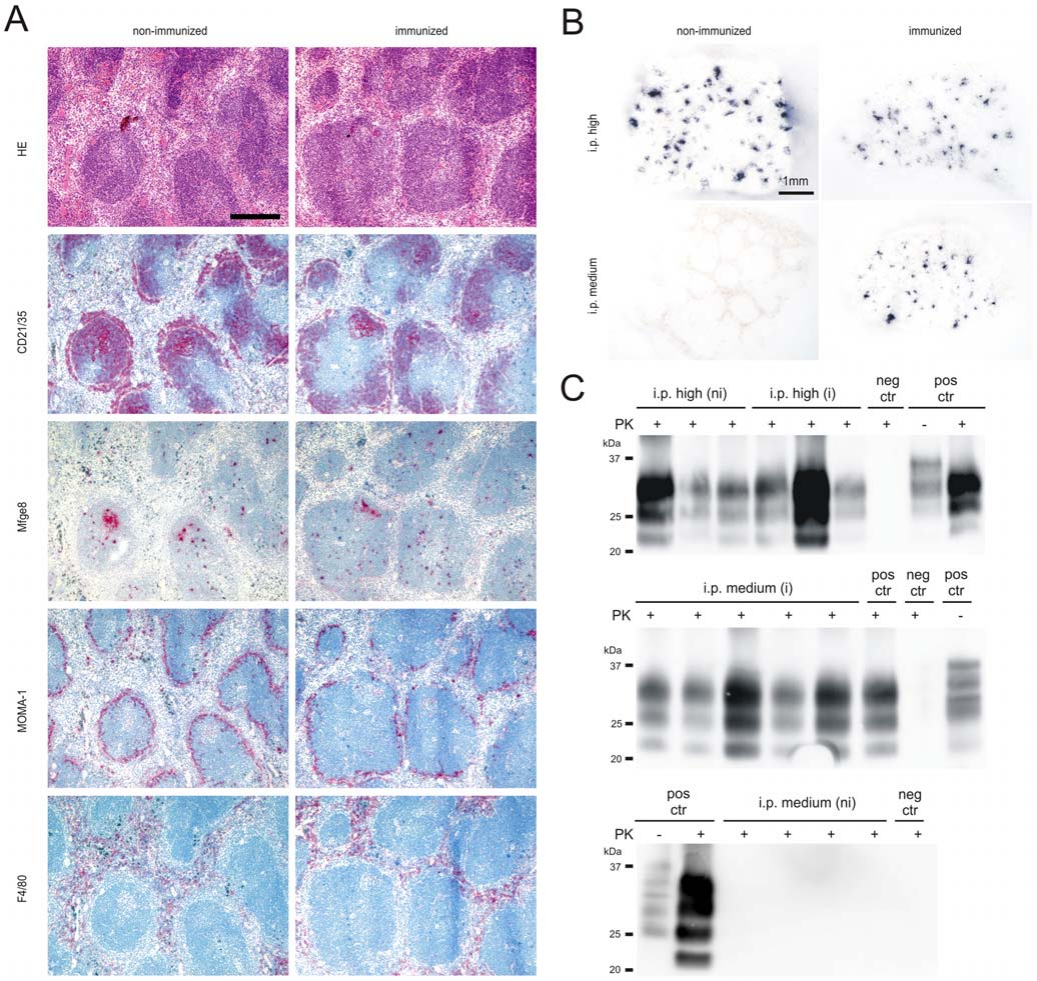
Histology, histoblots, and Western blots of spleens from terminally sick or asymptomatic mice. (A) Spleens of immunized (i) and non-immunized mice (ni) inoculated intraperitoneally (i.p.) with medium or high dose of prions were analyzed by histology. Histological and immunohistochemical stains were performed as indicated. MOMA-1^+^ cells were reduced in immunized mice. All terminally sick mice of the various experimental groups carried PrP^Sc^ in their spleens as shown by histoblot (B) and confirmed by Western Blot (C). Asymptomatic, non-immunized mice inoculated i.p. with a medium dose of prions [i.p. medium (ni)] were killed at 400 dpi. No PrP^Sc^ was detectable by histoblot analysis (B) and Western blot analysis (C). Controls and abbreviations are as in Fig. 6. Scale bars: histoblot = 1 mm; histology = 200 µm.

Similarly, spleens of terminally sick mice were investigated histologically and biochemically by histoblot and immunoblot. There was no difference in splenic histology of immunized mice compared to control (Fig. 7A), and PrP^Sc^ deposition pattern (Fig. 7B). Besides, we could not detect an overt difference in the PrP^Sc^ deposition as determined by immunoblot (Fig. 7C). As in brains, PrP^Sc^ was not detectable in spleens of healthy non-immunized mice at 400 dpi (n = 4) that had received 10 ng RML6 brain homogenate i.p. (Fig. 7B and 7C).

### No difference in splenic PrP^Sc^ deposition at 70 dpi

At 70 dpi there was no difference in PrP^Sc^ load in spleens of immunized mice vs. non-immunized controls. PrP^Sc^ was detectable after sodium phosphotungstic acid (NaPTA) precipitation in spleens of 1 of 4 immunized mice and of 1 of 4 non-immunized mice (Supplemental Figure S3A). The early presence of PrP^Sc^ in spleens (at 70 dpi) did not correlate with the observed difference in prion susceptibility. PrP^Sc^ was below the limit of detection of the NaPTA immunoblot technique in mesenteric lymph nodes of non-immunized and immunized mice at 70 dpi (Supplemental Figure S3A). Since it was previously reported that immune stimulation might increase expression levels of PrP^C^ on blood cells [54], we further analyzed PrP expression and cellular blood composition by flow cytometry analysis. There was no significant increase of PrP on CD19^+^ B-cells, CD3^+^ T-cells, or CD11b^+^/CD11c^+^ monocytes in immunized vs. non-immunized mice. However, the overall PrP expression on all live gated blood cells was slightly increased in immunized vs. non-immunized mice, but this trend was not significant (immunized: 30.4±2.81% of PrP positive white blood cells; non-immunized: 25.3±1.57%; unpaired t-test *p* = 0.12, values in mean ± standard error of the mean; Supplemental Figure S3B and data not shown).

We did observe a significant increase in PrP signal on live-gated blood cells in prion infected mice (immunized and non-immunized: 27.9±1.6% of PrP positive white blood cells) compared to non-inoculated, naive mice (16.6±1.8%; unpaired t-test p<0.002; Supplemental Figure S3B and data not shown). This rise in PrP signal can either be attributed to increased protein expression, decreased turn-over, or possibly accumulation of PrP^Sc^ on blood cells. As previously shown by hematology, there was no significant difference in the percentage of blood lymphocytes. However, there was a trend towards an increase of CD19^+^ B-cells in immunized, vs. non-immunized mice (Supplemental Figure S3B).

## Discussion

The outcome of encounters between a pathogen and its host is determined by two sets of parameters: the intrinsic virulence of the pathogen and the susceptibility of the host. Susceptibility is a measure of the likelihood to contract disease after exposure to a defined inoculum of a given pathogen. In addition to host-intrinsic modifiers, susceptibility to most pathogens is profoundly influenced by exogenous cofactors. Whereas immunodeficiencies greatly increase susceptibility to most conventional pathogens, theoretical considerations prompted us to investigate whether susceptibility to prions may be enhanced by stimulation of the immune system. Indeed, we have identified controlled, repetitive immunization as an important host factor that dramatically increases the susceptibility to peripherally administered prions. Following injection of a low dose of prions, 50% of the immunized mice developed scrapie while all non-immunized mice remained scrapie-free for >500 dpi.

In most instances the likelihood of survival after exposure to infectious agents or poisons is normally distributed, and therefore cumulative dose-survival curves display sigmoidal responses with subthreshold doses failing to elicit disease and high doses plateauing towards maximal effects. As a consequence, variations in host susceptibility are typically visible only within a limited range of doses. Here we tested three different doses of prions in immunized and non-immunized mice. The lowest dose led to 100% survival, whereas the highest dose led to 12.5% (i.p.) and 0% (i.c.) survival, in both groups. The medium dose of intraperitoneally administered prions was associated with an attack rate of 50% in immunized mice only, which nominally corresponds to one LD50 unit (dose causing death in 50% of the exposed hosts) and to the turning point of the postulated sigmoid response curve. Therefore, in this paradigm the medium dose allowed for optimal sensitivity in the identification of immunization as a factor altering prion susceptibility.

Treatment with complete Freund's adjuvant (CFA) was reported to prolong survival of prion-inoculated mice, irrespective of whether the challenge was i.c. or i.p. [55]. These results implied that CFA treatment might have a therapeutic effect in experimental scrapie, possibly by reducing the rate of PrP^Sc^ accumulation in the brain. In our experiments we did not detect any influence on susceptibility to i.c. administered prions, and a sensitizing effect after peripheral prion administration. Possible explanations for this discrepancy are (1) the different agents and protocols (site of injection, frequency) used for immunization and (2) the different titers of prion inocula applied.

Another study reported that transient systemic inflammation induced by i.p. administration of LPS acutely exacerbates cognitive and motor symptoms after inoculation with the mouse-adapted ovine prion strain Me7 and accelerates disease progression [56]. Accordingly, we found that immunized mice inoculated i.c. with prions became terminally sick slightly earlier than controls. Although we investigated the influence of other immunizing agents on another prion strain, our finding might well be explained by an acceleration of disease progression as suggested [56]. In contrast, we focused on differences in disease susceptibility rather than disease onset and progression.

Another study investigated the impact on experimental autoimmune encephalomyelitis (EAE) on prion susceptibility and incubation time. Increased prion susceptibility was observed after low dose i.c. inoculation [57], whereas we could not observe any change in prion susceptibility after low dose i.c. inoculation. This may point to additional EAE-related effects on prion transport, such as e.g. disturbance of the blood-brain barrier.

It was previously reported that susceptibility of mice to BSE and RML5 prions is not affected by the activation of dendritic cells [49]. Also, we previously found that chronic lymphofollicular inflammation of kidney, pancreas, and liver did not overtly alter susceptibility [34]. However, in all of these studies the scrapie attack rates were 100% in both the experimental and the control groups of mice, suggesting that the size of the prion inocula have precluded the sensitive detection of susceptibility shifts. This interpretation is congruent with our finding of indistinguishable attack rates in non-immunized and immunized mice peripherally exposed to a relatively large inoculum (300 ng of brain homogenate).

It had been previously reported that repeated injection of CpG-ODN protects mice from prion infections [50], suggesting beneficial effects of immune hyperactivity on prion susceptibility, but this treatment was subsequently found to disturb microarchitecture and functionality of lymphoid organs [51]. Therefore, the interpretation of the current findings crucially relies on the intactness of the immune system. Indeed, histology, hematology, and serum chemistry failed to identify immuno-suppressive consequences of the herein described treatment. Our data add weight to the conjecture that immunization increases susceptibility to prions and that the previously described protective effects were caused by immuno-disruption rather than by stimulation of the immune system.

As a consequence of the immune stimulation protocol, we observed moderate hepatitis. This might be explained by the hepatotoxic effects of IL-6, which was increased in serum of immunized mice as previously reported [58]. The increased susceptibility of immunized mice was restricted to peripheral prion exposure, and was not observed after i.c. prion inoculation. Evidently immune modulation appears to affect the speed and/or the extent of prion replication outside but not within the CNS. This suggests that CNS cells are constitutively competent for prion replication, whereas peripheral sites (which may comprise not only lymphoid compartments but also e.g. peripheral nerves) appear to be susceptible to immunological modifiers.

The precise mechanisms by which repetitive immunization increases prion susceptibility remain unknown. Any of the steps involved in peripheral prion spread, including transfer of prions to lymphoid tissues, prion accumulation and propagation within lymphoid tissues, and prion transfer from lymphoid tissues to structures of the nervous system, might be affected by immunization.

The kinetics of PrP^Sc^ accumulation in lymphatic organs might arguably play an important role in susceptibility to peripheral prion administration. While all terminally sick mice carried PrP^Sc^ in the spleen, PrP^Sc^ was undetectable in spleens of all mice that remained healthy. At early time points (70 dpi), before neuroinvasion had taken place [17], there was no difference in splenic PrP^Sc^ load. Hence, chronic inflammation determines the susceptibility towards prions by a mechanism that is independent of the early splenic PrP^Sc^ accumulation. Also, the absence of PrP^Sc^ from MLNs at 70 dpi suggests that at least in early stages of disease, and under the conditions studied here, MLNs do not play an essential role in prion pathogenesis.

The absence of PrP^Sc^ from spleens at ≥400 dpi despite its presence at earlier stages suggests the existence of a splenic prion clearance mechanism. Maybe repetitive immunization of the immune system facilitates neuroinvasion by reducing splenic prion clearance. The reduced numbers of MOMA-1^+^ marginal zone macrophages in immunized mice could contribute to an inhibited prion clearance. MOMA-1^+^ cells play an important role in capturing blood borne antigens [59]. Involvement of splenic macrophages in prion clearance in the early phase of infection has been reported, and it was even proposed that marginal zone macrophages are precisely involved in this process [60]. This suggests that a decreased prion uptake by MOMA-1^+^ cells might lead to a more efficient prion translocation into germinal centers in immunized mice in our study. In this context, it has previously been suggested that CpG-ODN treatment inhibits prion clearance by microglia cells in vitro [54].

We have found an increase in marginal zone B-cells in immunized mice. It is conceivable that alterations in the number of marginal zone B-cells in combination with a decreased number of MOMA-1^+^ cells might enhance prion uptake into the germinal centers, thereby influencing prion susceptibility. APCs were proposed to either support prion clearance or to accomplish prion transport [44], [45], [46], [47], [48], [49]. Immunohistochemical as well as flow cytometry analysis of blood and spleen from immunized mice did not reveal a significant change of CD11c^+^ or CD11b^+^ APCs. Therefore, it is very unlikely that quantitative changes in APCs would contribute to the observed increase in prion susceptibility.

FDCs play an important role in prion replication and are known to co-localize with PrP^Sc^. In our study, there was an increase in size and density of FDC networks following immunization. This might well explain the increased susceptibility in immunized mice. Alternatively, chronic stimulation of the immune system may alter the transfer of prions from lymphoid organs to the nervous system. This transfer has previously been shown to be rate-limiting in prion pathogenesis, involving FDCs within germinal centers and tyrosine hydroxylase (TH) positive sympathetic nerve endings. Since immune activation can alter splenic innervation [61], we wondered whether the distance between FDCs and terminal nerve endings was changed, contributing to the increased prion susceptibility of immunized mice. However, staining for tyrosine hydroxylase failed to show altered distances between nerve endings and follicles (data not shown).

Altered PrP^C^ levels in secondary lymphoid organs can potentially impact prion susceptibility or prion incubation time. Immunization in this study did not alter the overall splenic PrP^C^ levels as determined by ELISA. Interestingly, by flow cytometry, a slight increase in PrP^C^ cell surface expression was detected on splenic B- and CD4^+^ T-cells in immunized mice. We cannot exclude that this slightly increased PrP^C^ expression on either of the two cell types contributes to increased susceptibility. However, previous studies have shown that PrP^C^ neither on B- nor on T-cells alone suffices for efficient prion replication [18], [62]. Therefore, we presume that PrP^C^ levels on B- and T-cells are not the susceptibility determining factors in our study.

CpG-ODN and LPS treatment can increase the cell surface level of PrP^C^ on macrophages, and it has been suggested that macrophages can replicate prions transiently after stimulation with LPS or CpG-ODN [54]. The conditions used here for immunization did not significantly increase PrP levels on white blood cells or splenic CD11c^+^ or CD11b^+^ cells. Therefore, it is unlikely that PrP levels on white blood cells or antigen presenting cells within the spleen determined prion susceptibility in our study.

We have found an increased density of splenic granulocytes following immunization. This might have an impact on prion susceptibility. Since the role of granulocytes in prion pathogenesis is unknown, no definite conclusions can be drawn. Similarly, the impact of CD68^+^ cells appearing in the white pulp follicles of immunized mice and its effect on peripheral prion replication remain unknown. Alum, which was used as an immunization adjuvant in our study, was recently shown to induce influx of monocytic dendritic cells into the peritoneum. This Nalp3-dependent influx [63] may conceivably represent a mechanism for efficient transfer of prions to secondary lymphoid organs. It will be interesting to test whether interference with this pathway modulates prion susceptibility.

In summary, we conclude that either (1) increased density and size of FDC networks in splenic germinal centers, (2) decreased clearance of prions by changing the cellular compartment of the marginal zone (e.g. marginal zone macrophages, marginal zone B-cells), or (3) both, are most likely responsible for increased prion susceptibility upon immunization. In the light of the above results, it will be of interest to determine whether stimulation of the immune system may contribute to the individual susceptibility of humans towards prions. States of chronically stimulated immune system are common in humans, and can occur e.g. in chronic infectious diseases, autoimmune disorders, and allergies. Exposure to BSE prions is the assumed cause of vCJD, yet it is not known which factors determined that some unfortunate individuals among the European population developed vCJD while most others remained healthy. Epidemiological retrospective and prospective studies may help clarifying whether particular immune stimuli correlate with the likelihood to contract vCJD or other acquired prion diseases.

## Materials and Methods

### Mice and scrapie inoculation

Female wild-type (C57BL/6) mice were obtained from Harlan, NL and maintained under specific pathogen-free (SPF) conditions. Housing and experimental protocols were in accordance with the Swiss Animal Protection Law and mice were held in compliance with the regulations of the Veterinäramt, Kanton Zürich. Mice were infected intraperitoneally (i.p.) with 100 µl brain homogenate derived from terminally scrapie sick CD-1 mice, homogenized in PBS/0.32M sucrose, or intracerebrally (i.c.) with 30 µl brain homogenate. Different doses of prions derived from a RML6 infected terminally sick wild-type mouse were injected intraperitoneally (i.p.) or intracerebrally (i.c.) (i.p. doses: 0.33 ng, 10 ng, 300 ng; i.c. doses: 10 pg, 300 pg, 9 ng brain homogenate). Mice were euthanized at 0, 70 and 400 dpi or when terminally scrapie sick.

### Repetitive immunization

Repetitive immunization was performed in female wild-type (C57BL/6) mice by repeated injections of CpG-ODN and BSA/alum. BSA was diluted in 200 µl of freshly prepared Al(OH)_3_ to a final concentration of 50 µg BSA per injection per mouse as described [43]. Intraperitoneal injections of either 30 µg CpG-ODN (Coley pharma, ODN 1826) or BSA/alum were performed as indicated in Fig. 1A, starting 6 weeks prior to prion inoculation.

### Western blot analysis and NaPTA precipitation

Tissue homogenates (brain; spleen) were adjusted to 8 mg/ml protein, and treated with proteinase K (20–50 µg/ml, 30 min, 37°C). 50 µg of total protein was loaded onto a NuPAGE® Novex 12% Bis-Tris Gel (Invitrogen) and separated. Proteins were then transferred to nitrocellulose membrane (see below). For detection of PrP^Sc^ in spleen homogenates NaPTA (sodium phosphotungstic acid) precipitation was performed. 10% spleen homogenates were prepared in PBS on ice. Cellular debris was removed by centrifugation at 500 g for 1–2 min. The resulting supernatant was adjusted to 500 µl with PBS, and mixed 1∶1 with 4% Sarkosyl in PBS. Samples were incubated for 15 min at 37°C under constant agitation. Benzonase and MgCl_2_ were added to a final concentration of 50 U/ml and 1 mM respectively, and incubated for 30 min at 37°C under continuous agitation. Further, samples were digested with 30 µg/ml proteinase K (PK) for 60 min at 37°C with agitation and pre-warmed NaPTA stock solution (pH 7.4) was added to a final concentration of 0.3% and the sample was incubated at 37°C for 30 min with shaking, followed by centrifugation at 37°C for 30 min at 14.000 g in an Eppendorf microcentrifuge. The pellet was resuspended in 30 µl 0.1% Sarkosyl in PBS and the sample was heated at 95°C for 5 min in SDS-containing loading buffer before loading onto NuPAGE® Novex 12% Bis-Tris Gel (Invitrogen). All gels were transferred to nitrocellulose (Schleicher & Schuell) using XCell II Blot Module (Invitrogen). Membranes were blocked with TBST containing 5% non-fat milk, decorated with monoclonal antibody POM1 [64], [65] followed by incubation with the secondary anti-mouse IgG_1_ (Zymed) and visualized by enhanced chemiluminescence (ECL, Socochim, Pierce).

### Histology, immunohistochemistry, and histoblot

Paraffin sections (2 µm) and frozen sections (5 or 10 µm) of various organs were stained with hematoxylin-eosin. Antibodies raised against the following antigens were used for immunohistochemistry: FDC-M1 (Mfge8) for mature FDCs (clone 4C11; 1∶50; Becton Dickinson), B220/CD45R for B-cells (Pharmingen; 1∶400), CD35 for CR1 (clone 8C12, Pharmingen, San Diego, CA; 1∶100), CD3 for T-cells (clone SP7, NeoMarkers; 1∶300), F4/80 for macrophages (Serotec; 1∶50), PNA for germinal center B-cells (Vector L-1070; 1∶100), MOMA-1 for metallophilic marginal zone macrophages (BMA, Augst, Swizerland; 1∶50), GFAP for astrocytes (DAKO, Carpinteris, CA; 1∶300), and Iba-1 for microglia (WAKO; 1∶2500).

PrP stains were performed on formalin-fixed brain tissues treated with concentrated formic acid to inactivate prions and postfixed again in formalin. Subsequently, tissues were embedded in paraffin. After deparaffination, sections (2 µm) were incubated for 6 min in 98% formic acid and washed in distilled water for 30 min. Sections were heated to 100°C in a steamer in citrate buffer (pH 6.0) for 3 min, and allowed to cool down to room temperature. Sections were incubated in Ventana buffer and stains were performed on a NEXES immunohistochemistry robot (Ventana instruments, Switzerland) using an IVIEW DAB Detection Kit (Ventana). After incubation with protease 1 (Ventana) for 16 min, sections were incubated with anti-PrP SAF-84 (SPI bio; 1∶200) for 32 min. Sections were counterstained with hematoxylin. Histoblot analysis was performed as described [66]. Image acquisition was performed on an Axiophot-microscope (Zeiss) equipped with a JVC digital camera (KY-F70; 3CCD).

### In situ hybridization

Digoxigenin (DIG)-labeled *Mfge8* riboprobe was obtained by transcription of pBluescript II KS+ (Stratagene) containing the open reading frame of *Mfge8 and using a DIG RNA labeling kit (Roche)*. ISH was performed on spleen cryosections. Sections were fixed in 4% paraformaldehyde PBS, followed by acetylation. After prehybridization, 200 ng/ml of DIG-labeled RNA probe was added to the hybridization buffer and incubated at 72°C overnight. DIG-labeled probes were detected by anti-DIG–alkaline phosphatase Fab-fragments (Roche). Cell nuclei were stained with DAPI (4′,6-Diamidine-2′-phenylindole dihydrochloride; Roche).

### RNA isolation from spleen and real-time PCR analysis

RNA isolation buffer (RLT; Qiagen) was added to flash frozen spleens prior to homogenization (Medic tools). RNA was purified using RNeasy (Qiagen) as described by the manufacturer. Synthesis of cDNA was performed with QuantiTect, Reverse Transcription kit (Qiagen). Samples were analyzed by real-time PCR using QuantiFast SYBR Green PCR kit (Qiagen) and 7900HT (Fast Real-Time PCR systems; Applied Biosystems). The following primer combinations were used [forward primer (FW), reverse primer (RV)]: Mfge8 FW: 5′-ATA TGG GTT TCA TGG GCT TG-3′; Mfge8 RV: 5′-GAG GCT GTA AGC CAC CTT GA-3′; GAPDH FW: 5′-CCA CCC CAG CAA GGA GAC-3′; GAPDH RV: 5′- GAA ATT GTG AGG GAG ATG CT-3′

### PrP^C^ sandwich ELISA

96-well plates were coated with 20 ng of purified POM1 antibody overnight at 4°C. Plates were washed with PBS containing 0.1% (vol/vol) Tween 20 (PBST), and blocked with 5% Top-Block (Fluka) in TBST for 2 h at room temperature (RT). After washing, plates were incubated with 50 µl of spleen homogenates containing 500 µg/ml total protein in sample buffer (1% Top block in PBST). The total protein concentrations of the spleen homogenates were determined using a standard colorimetric assay based on bicinchoninic acid (BCA, Pierce). Each sample was analyzed in triplicates. For the standard curve, serially diluted recombinant mouse PrP23-230 in PBST containing 1% Top Block was used. After 1 h at RT plates were washed extensively and then probed with biotinylated POM2 [64], [65] at a concentration of 200 ng/ml in PBST containing 1% Top Block, for 1 h at room temperature. After washing, plates were incubated with horseradish peroxidase conjugated Avidin (1∶1000 dilution, BD-Pharmingen) for 1 h at RT. Plates were developed with Stabilized Chromogen SB02 (Biosource). The chromogenic reaction was stopped by adding the same volume of 0.5 M H_2_SO_4_. Optical density was measured at 405 nm. The PrP^C^ concentration in each sample was calculated according to a standard curve derived from the values of recombinant PrP.

### Fluorochrome labeling

1 mg of Cy5 NHS ester (Amersham Biosciences; Cat. No. PA15101) was dissolved in 1 ml (conc. 1 mg/ml) water-free DMSO. 1 ml of the POM2 antibody [64], [65] (conc. 2 mg/ml) and of 0.5 M borate buffer (pH 8.0) leading to an antibody concentration of 1.81 mg/ml. 2 mg POM2 (1.1 ml) were used for the labelling reaction (Dye/antibody ratio = 2.5/1). 2 mg POM2 were mixed with 24.80 ml of Cy5 dye (31.25 nmol). The Cy5 dye was added to the antibody while vortexing and further on incubated under permanent agitation for 1 h at RT (light protected). The unincorporated Cy5 dye was removed in a size-exclusion column and the labeled antibody was dialyzed against 2 l of PBS over night.

### Flow cytometric analysis

Splenic cells or blood cells were isolated and incubated with primary antibodies: POM2-Cy5 (1∶250), FITC- or PE-labeled anti-CD11b antibody, FITC- or PE-labeled anti-CD11c antibody, PE-labeled anti-CD19 antibody, FITC-labeled anti-B220 antibody, PE-labeled anti-Ly6G antibody, PE-labeled anti-CD8, PE-labeled anti-CD3 antibody (all 1∶100, Pharmingen), PerCP-labeled anti-CD4 (1∶750), or FITC-labeled anti-CD21 and PE-labeled anti-CD23 (1∶100 and 1∶250, respectively). Erythrocyte lysis was performed using BD FACS^TM^ Lysing Solution. Cells were washed with PBS containing 2% FCS and live gated blood cells were further analyzed with a flow cytometer (DAKO or BD Biosciences) and FlowJow software program.

## Supporting Information

Figure S1 In situ hybridization for *Mfge8* mRNA. Spleens of non-immunized and immunized mice were analyzed at the time point of inoculation by in situ hybridization for *Mfge8* mRNA. Consecutive sections were hybridized with *Mfge8* antisense (AS) and control sense probe (S) as well as DAPI stains. Scale bar = 100 µm.
(7.90 MB TIF)

Figure S2 Histology of mesenteric lymph nodes, various organs, and peritoneum of immunized and non-immunized mice. (A) Mesenteric lymph nodes (MLNs) were analyzed at the time point of inoculation by histology and immunohistochemistry. Stains and immunohistochemistry were performed as indicated: HE, B-cells (B220), germinal center B-cells and FDCs (CD21/35), FDCs (Mfge8), germinal centers (PNA) and metallophilic marginal zone macrophages (MOMA-1). (B) Additional organs were analyzed. While lung, kidney, and pancreas displayed a normal architecture, liver showed lobular hepatitis with loss of hepatocytes and multifocal infiltrates of T-lymphocytes (CD3), macrophages (F4/80), eosinophils, and neutrophils. (C) Immunized mice showed infiltrates of T-cells (CD3) and macrophages (F4/80) in the peritoneum, demonstrating peritonitis. All scale bars = 100 µm.
(7.43 MB TIF)

Figure S3 PrP in spleens, mesenteric lymph nodes (MLNs), and on blood cells of inoculated mice at 70 dpi. Mice were inoculated i.p. with a medium dose of prions and analyzed at 70 dpi. (A) NaPTA-enhanced Western blots of spleen and MLN homogenates. We detected PrP^Sc^ in spleens of 1 of 4 mice in both, the immunized (i) and non-immunized (ni) group. Controls and abbreviations are as in Fig. 6. No PrP^Sc^ deposition was detectable in MLNs of the same mice by NaPTA-enhanced Western blotting. (B) Flow cytometry analysis of white blood cells from immunized and non-immunized mice inoculated i.p. with prions, as well as non-inoculated, non-immunized naive mice. Co-staining for PrP (Cy5-labeled POM2 antibody) and CD19 (PE-labeled anti-CD19 antibody) in representative samples. Four mice per group were analyzed. Numbers in the diagram indicate averages (as percentages) ± standard deviation. Blood from a PrP deficient mouse (*Prnp^−/−^*) served as negative control.
(1.01 MB TIF)

## References

[1] Collinge J (2005). Molecular neurology of prion disease.. J Neurol Neurosurg Psychiatry.

[2] Kovacs GG, Budka H (2008). Prion diseases: from protein to cell pathology.. Am J Pathol.

[3] Frigg R, Klein MA, Hegyi I, Zinkernagel RM, Aguzzi A (1999). Scrapie pathogenesis in subclinically infected B-cell-deficient mice.. J Virol.

[4] Aguzzi A, Haass C (2003). Games played by rogue proteins in prion disorders and Alzheimer's disease.. Science.

[5] Aguzzi A, Baumann F, Bremer J (2008). The Prion's Elusive Reason for Being.. Annu Rev Neurosci.

[6] Aguzzi A (2003). Prions and the immune system: a journey through gut, spleen, and nerves.. Adv Immunol.

[7] Aguzzi A, Sigurdson C, Heikenwalder M (2008). Molecular Mechanisms of Prion Pathogenesis.. Annu Rev Pathol.

[8] Collinge J, Whitfield J, McKintosh E, Beck J, Mead S (2006). Kuru in the 21st century—an acquired human prion disease with very long incubation periods.. Lancet.

[9] Collinge J (1999). Variant Creutzfeldt-Jakob disease.. Lancet.

[10] Collinge J, Rossor M (1996). A new variant of prion disease.. Lancet.

[11] Aguzzi A (1996). Between cows and monkeys.. Nature.

[12] Aguzzi A, Weissmann C (1996). Spongiform encephalopathies: a suspicious signature.. Nature.

[13] Bruce ME, Will RG, Ironside JW, McConnell I, Drummond D (1997). Transmissions to mice indicate that ‘new variant’ CJD is caused by the BSE agent.. Nature.

[14] Hill AF, Desbruslais M, Joiner S, Sidle KC, Gowland I (1997). The same prion strain causes vCJD and BSE.. Nature.

[15] Lasmezas CI, Deslys JP, Demaimay R, Adjou KT, Lamoury F (1996). Bse Transmission to Macaques.. Nature.

[16] Büeler HR, Fischer M, Lang Y, Bluethmann H, Lipp HP (1992). Normal development and behaviour of mice lacking the neuronal cell-surface PrP protein.. Nature.

[17] Büeler HR, Aguzzi A, Sailer A, Greiner RA, Autenried P (1993). Mice devoid of PrP are resistant to scrapie.. Cell.

[18] Raeber AJ, Sailer A, Hegyi I, Klein MA, Rülicke T (1999). Ectopic expression of prion protein (PrP) in T lymphocytes or hepatocytes of PrP knockout mice is insufficient to sustain prion replication.. Proc Natl Acad Sci U S A.

[19] Raeber AJ, Klein MA, Frigg R, Flechsig E, Aguzzi A (1999). PrP-dependent association of prions with splenic but not circulating lymphocytes of scrapie-infected mice.. EMBO J.

[20] Collinge J, Beck J, Campbell T, Estibeiro K, Will RG (1996). Prion Protein Gene Analysis in New Variant Cases of Creutzfeldt-Jakob Disease.. Lancet.

[21] Zeidler M, Stewart GE, Barraclough CR, Bateman DE, Bates D (1997). New variant Creutzfeldt-Jakob disease: neurological features and diagnostic tests.. Lancet.

[22] Hill AF, Butterworth RJ, Joiner S, Jackson G, Rossor MN (1999). Investigation of variant Creutzfeldt-Jakob disease and other human prion diseases with tonsil biopsy samples.. Lancet.

[23] Shibuya S, Higuchi J, Shin RW, Tateishi J, Kitamoto T (1998). Codon 219 Lys allele of PRNP is not found in sporadic Creutzfeldt-Jakob disease.. Ann Neurol.

[24] Matthews L, Coen PG, Foster JD, Hunter N, Woolhouse ME (2001). Population dynamics of a scrapie outbreak.. Arch Virol.

[25] Ferguson NM, Donnelly CA, Woolhouse ME, Anderson RM (1997). The epidemiology of BSE in cattle herds in Great Britain. II. Model construction and analysis of transmission dynamics.. Philos Trans R Soc Lond B Biol Sci.

[26] Boelle PY, Cesbron JY, Valleron AJ (2004). Epidemiological evidence of higher susceptibility to vCJD in the young.. BMC Infect Dis.

[27] Outram GW, Dickinson AG, Fraser H (1974). Reduced susceptibility to scrapie in mice after steroid administration.. Nature.

[28] Kitamoto T, Muramoto T, Mohri S, Doh ura K, Tateishi J (1991). Abnormal isoform of prion protein accumulates in follicular dendritic cells in mice with Creutzfeldt-Jakob disease.. J Virol.

[29] Mabbott NA, Williams A, Farquhar CF, Pasparakis M, Kollias G (2000). Tumor necrosis factor alpha-deficient, but not interleukin-6-deficient, mice resist peripheral infection with scrapie.. J Virol.

[30] Prinz M, Montrasio F, Klein MA, Schwarz P, Priller J (2002). Lymph nodal prion replication and neuroinvasion in mice devoid of follicular dendritic cells.. Proc Natl Acad Sci U S A.

[31] Montrasio F, Frigg R, Glatzel M, Klein MA, Mackay F (2000). Impaired prion replication in spleens of mice lacking functional follicular dendritic cells.. Science.

[32] Mabbott NA, Mackay F, Minns F, Bruce ME (2000). Temporary inactivation of follicular dendritic cells delays neuroinvasion of scrapie.. Nat Med.

[33] Mabbott NA, Young J, McConnell I, Bruce ME (2003). Follicular dendritic cell dedifferentiation by treatment with an inhibitor of the lymphotoxin pathway dramatically reduces scrapie susceptibility.. Journal of Virology.

[34] Heikenwalder M, Zeller N, Seeger H, Prinz M, Klohn PC (2005). Chronic lymphocytic inflammation specifies the organ tropism of prions.. Science.

[35] Ligios C, Sigurdson CJ, Santucciu C, Carcassola G, Manco G (2005). PrPSc in mammary glands of sheep affected by scrapie and mastitis.. Nat Med.

[36] Seeger H, Heikenwalder M, Zeller N, Kranich J, Schwarz P (2005). Coincident scrapie infection and nephritis lead to urinary prion excretion.. Science.

[37] Outram GW, Dickinson AG, Fraser H (1973). Developmental maturation of susceptibility to scrapie in mice.. Nature.

[38] Ierna M, Farquhar CF, Outram GW, Bruce ME (2006). Resistance of neonatal mice to scrapie is associated with inefficient infection of the immature spleen.. J Virol.

[39] Prinz M, Heikenwalder M, Junt T, Schwarz P, Glatzel M (2003). Positioning of follicular dendritic cells within the spleen controls prion neuroinvasion.. Nature.

[40] Glatzel M, Aguzzi A (2000). PrP(C) expression in the peripheral nervous system is a determinant of prion neuroinvasion.. J Gen Virol.

[41] Glatzel M, Heppner FL, Albers KM, Aguzzi A (2001). Sympathetic innervation of lymphoreticular organs is rate limiting for prion neuroinvasion.. Neuron.

[42] Raymond CR, Mabbott NA (2007). Assessing the involvement of migratory dendritic cells in the transfer of the scrapie agent from the immune to peripheral nervous systems.. J Neuroimmunol.

[43] Heikenwalder M, Federau C, von Boehmer L, Schwarz P, Wagner M, Zeller N, Haybaeck J, Prinz M, Becher B, Aguzzi A (2007). Germinal center B cells are dispensable in prion transport and neuroinvasion.. J Neuroimmunol.

[44] Cordier-Dirikoc S, Chabry J (2008). Temporary depletion of CD11c+ dendritic cells delays lymphoinvasion after intraperitonal scrapie infection.. J Virol.

[45] Levavasseur E, Metharom P, Dorban G, Nakano H, Kakiuchi T (2007). Experimental scrapie in ‘plt’ mice: an assessment of the role of dendritic-cell migration in the pathogenesis of prion diseases.. J Gen Virol.

[46] Mohan J, Bruce ME, Mabbott NA (2005). Neuroinvasion by scrapie following inoculation via the skin is independent of migratory Langerhans cells.. J Virol.

[47] Raymond CR, Aucouturier P, Mabbott NA (2007). In vivo depletion of CD11c+ cells impairs scrapie agent neuroinvasion from the intestine.. J Immunol.

[48] Aucouturier P, Geissmann F, Damotte D, Saborio GP, Meeker HC (2001). Infected splenic dendritic cells are sufficient for prion transmission to the CNS in mouse scrapie.. J Clin Invest.

[49] Dore G, Leclerc C, Lazarini F (2008). Treatment by CpG or Flt3-ligand does not affect mouse susceptibility to BSE prions.. J Neuroimmunol.

[50] Sethi S, Lipford G, Wagner H, Kretzschmar H (2002). Postexposure prophylaxis against prion disease with a stimulator of innate immunity.. Lancet.

[51] Heikenwalder M, Polymenidou M, Junt T, Sigurdson C, Wagner H (2004). Lymphoid follicle destruction and immunosuppression after repeated CpG oligodeoxynucleotide administration.. Nat Med.

[52] Aguzzi A, Sigurdson CJ (2004). Antiprion immunotherapy: to suppress or to stimulate?. Nat Rev Immunol.

[53] Kranich J, Krautler NJ, Heinen E, Polymenidou M, Bridel C (2008). Follicular dendritic cells control engulfment of apoptotic bodies by secreting Mfge8.. J Exp Med.

[54] Gilch S, Schmitz F, Aguib Y, Kehler C, Bulow S (2007). CpG and LPS can interfere negatively with prion clearance in macrophage and microglial cells.. Febs J.

[55] Tal Y, Souan L, Cohen IR, Meiner Z, Taraboulos A (2003). Complete Freund's adjuvant immunization prolongs survival in experimental prion disease in mice.. J Neurosci Res.

[56] Cunningham C, Campion S, Lunnon K, Murray CL, Woods JF (2008). Systemic Inflammation Induces Acute Behavioral and Cognitive Changes and Accelerates Neurodegenerative Disease.. Biol Psychiatry.

[57] Friedman-Levi Y, Ovadia H, Hoftberger R, Einstein O, Abramsky O (2007). Fatal neurological disease in scrapie-infected mice induced for experimental autoimmune encephalomyelitis.. J Virol.

[58] Naugler WE, Sakurai T, Kim S, Maeda S, Kim K (2007). Gender disparity in liver cancer due to sex differences in MyD88-dependent IL-6 production.. Science.

[59] Oehen S, Odermatt B, Karrer U, Hengartner H, Zinkernagel R (2002). Marginal zone macrophages and immune responses against viruses.. J Immunol.

[60] Beringue V, Demoy M, Lasmezas CI, Gouritin B, Weingarten C (2000). Role of spleen macrophages in the clearance of scrapie agent early in pathogenesis.. J Pathol.

[61] Yang H, Wang L, Huang CS, Ju G (1998). Plasticity of GAP-43 innervation of the spleen during immune response in the mouse. Evidence for axonal sprouting and redistribution of the nerve fibers.. Neuroimmunomodulation.

[62] Raeber AJ, Montrasio F, Hegyi I, Frigg R, Klein MA (2001). Studies on prion replication in spleen.. Developmental Immunology.

[63] Kool M, Petrilli V, De Smedt T, Rolaz A, Hammad H (2008). Cutting Edge: Alum Adjuvant Stimulates Inflammatory Dendritic Cells through Activation of the NALP3 Inflammasome.. J Immunol.

[64] Polymenidou M, Stoeck K, Glatzel M, Vey M, Bellon A (2005). Coexistence of multiple PrPSc types in individuals with Creutzfeldt-Jakob disease.. Lancet Neurol.

[65] Polymenidou M, Moos R, Scott M, Sigurdson C, Shi YZ (2008). The POM monoclonals: a comprehensive set of antibodies to non-overlapping prion protein epitopes.. PLoS ONE.

[66] Taraboulos A, Jendroska K, Serban D, Yang SL, DeArmond SJ (1992). Regional mapping of prion proteins in brain.. Proc Natl Acad Sci U S A.

