# Supplementary figures and images for: Repetitive Immunization Enhances the Susceptibility of Mice to Peripherally Administered Prions
Source: PLoS One. 2009 Sep 25;4(9):e7160. doi: 10.1371/journal.pone.0007160 (PMC2744926; doi:10.1371/journal.pone.0007160)

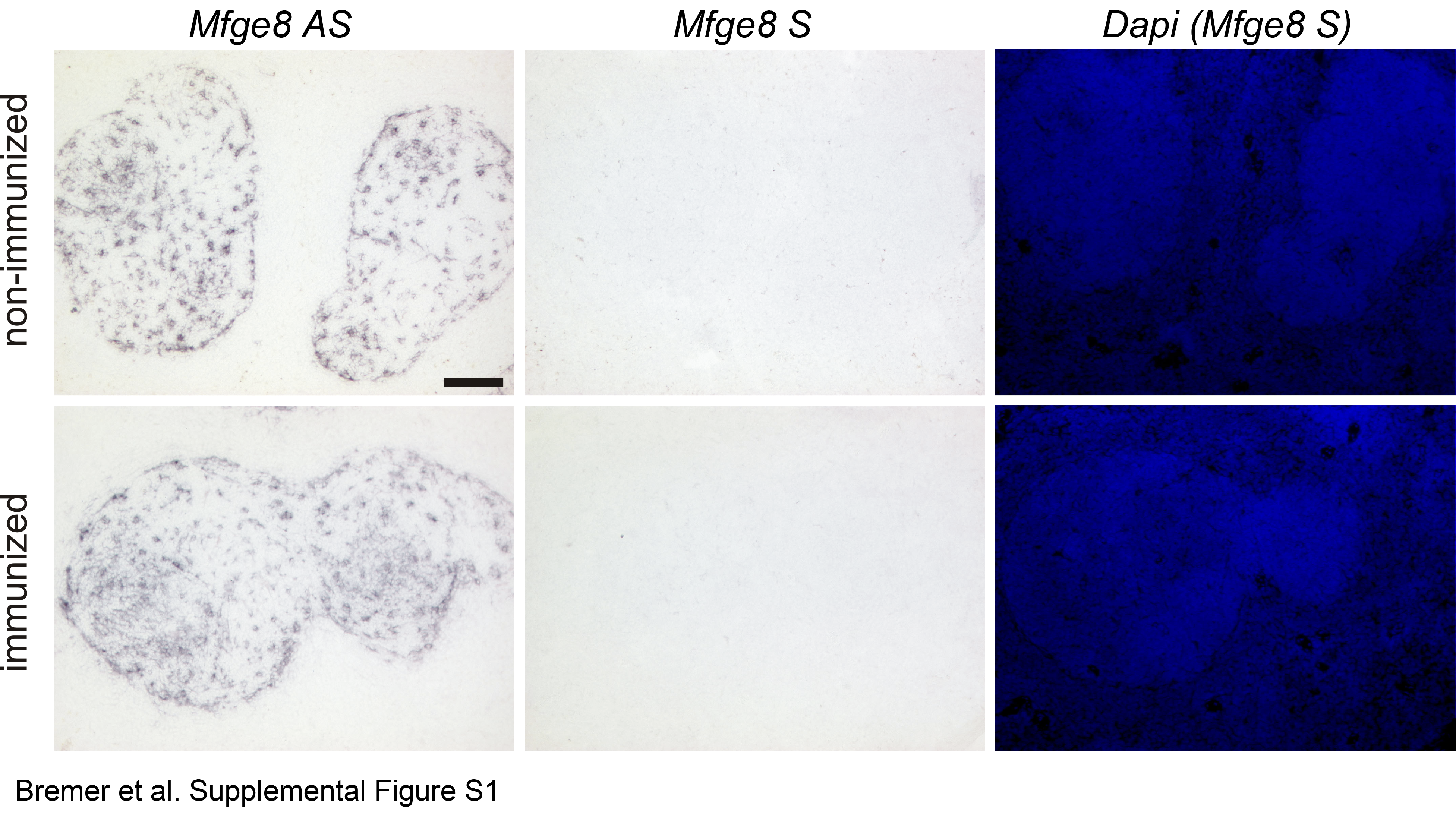

Supplement: Figure S1 — In situ hybridization for Mfge8 mRNA. Spleens of non-immunized and immunized mice were analyzed at the time point of inoculation by in situ hybridization for Mfge8 mRNA. Consecutive sections were hybridized with Mfge8 antisense (AS) and control sense probe (S) as well as DAPI stains. Scale bar = 100 µm. (7.90 MB TIF) [file pone.0007160.s001.tif]

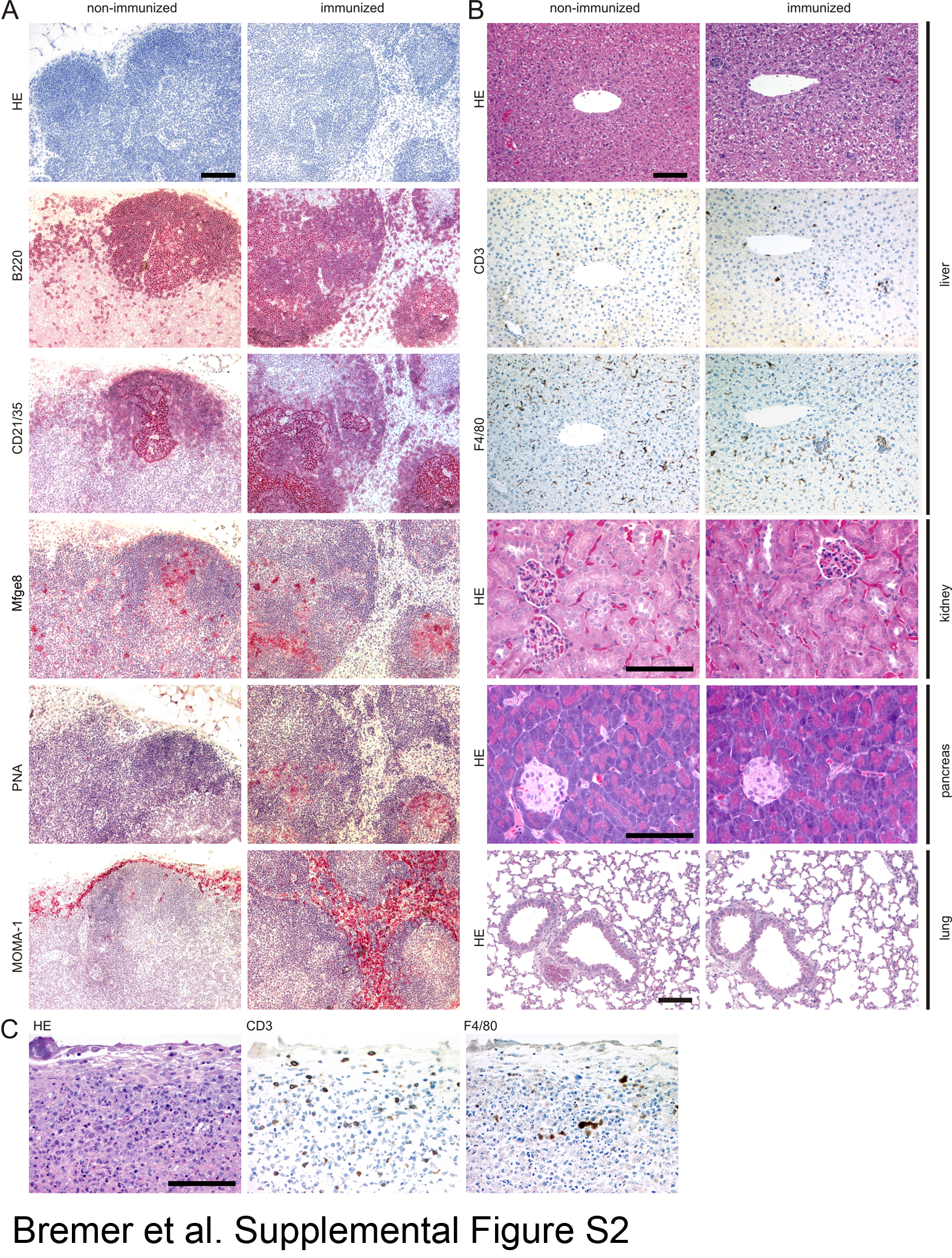

Supplement: Figure S2 — Histology of mesenteric lymph nodes, various organs, and peritoneum of immunized and non-immunized mice. (A) Mesenteric lymph nodes (MLNs) were analyzed at the time point of inoculation by histology and immunohistochemistry. Stains and immunohistochemistry were performed as indicated: HE, B-cells (B220), germinal center B-cells and FDCs (CD21/35), FDCs (Mfge8), germinal centers (PNA) and metallophilic marginal zone macrophages (MOMA-1). (B) Additional organs were analyzed. While lung, kidney, and pancreas displayed a normal architecture, liver showed lobular hepatitis with loss of hepatocytes and multifocal infiltrates of T-lymphocytes (CD3), macrophages (F4/80), eosinophils, and neutrophils. (C) Immunized mice showed infiltrates of T-cells (CD3) and macrophages (F4/80) in the peritoneum, demonstrating peritonitis. All scale bars = 100 µm. (7.43 MB TIF) [file pone.0007160.s002.tif]

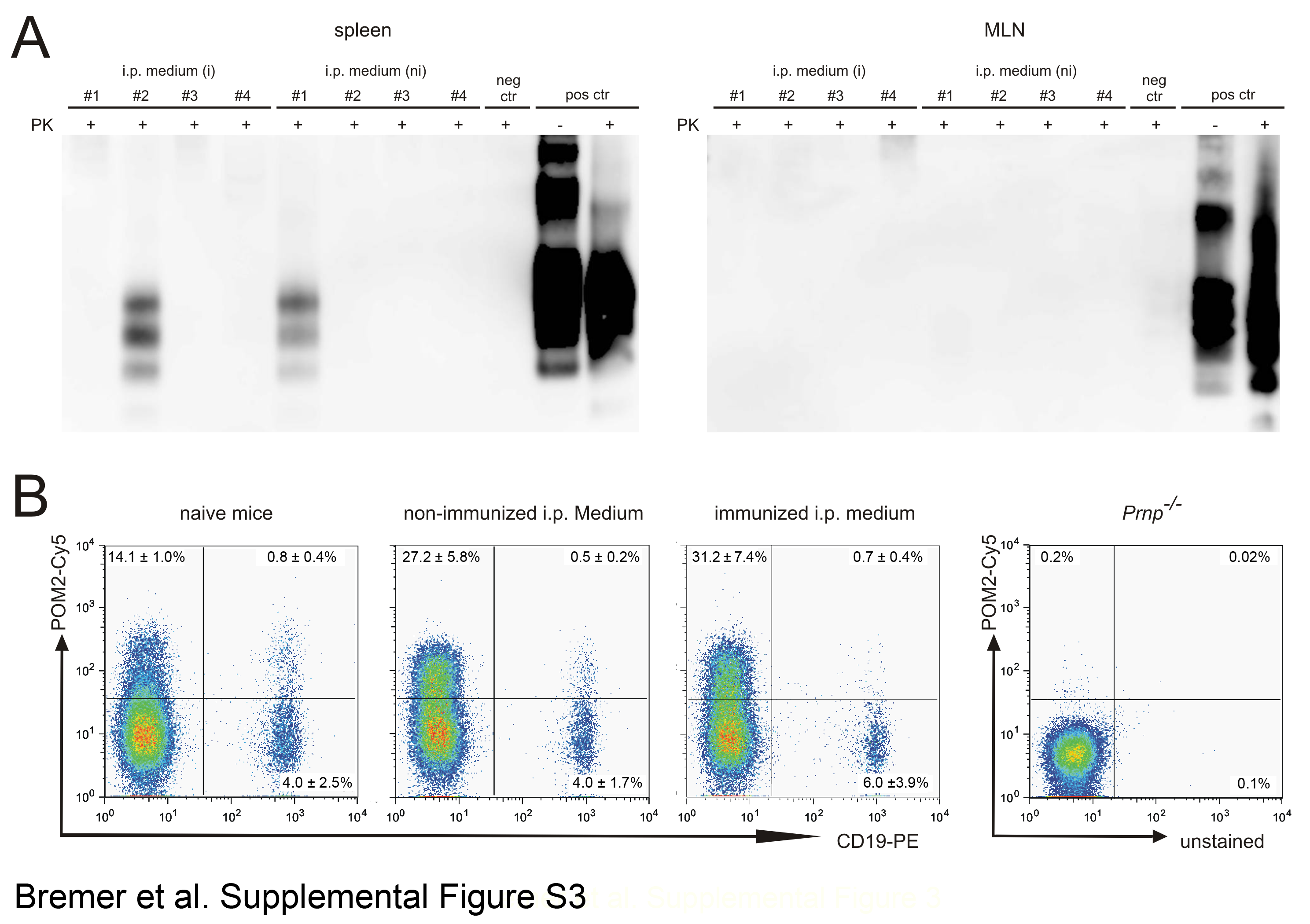

Supplement: Figure S3 — PrP in spleens, mesenteric lymph nodes (MLNs), and on blood cells of inoculated mice at 70 dpi. Mice were inoculated i.p. with a medium dose of prions and analyzed at 70 dpi. (A) NaPTA-enhanced Western blots of spleen and MLN homogenates. We detected PrPSc in spleens of 1 of 4 mice in both, the immunized (i) and non-immunized (ni) group. Controls and abbreviations are as in Fig. 6. No PrPSc deposition was detectable in MLNs of the same mice by NaPTA-enhanced Western blotting. (B) Flow cytometry analysis of white blood cells from immunized and non-immunized mice inoculated i.p. with prions, as well as non-inoculated, non-immunized naive mice. Co-staining for PrP (Cy5-labeled POM2 antibody) and CD19 (PE-labeled anti-CD19 antibody) in representative samples. Four mice per group were analyzed. Numbers in the diagram indicate averages (as percentages) ± standard deviation. Blood from a PrP deficient mouse (Prnp−/−) served as negative control. (1.01 MB TIF) [file pone.0007160.s003.tif]
